# Ketoanalogs’ Effects on Intestinal Microbiota Modulation and Uremic Toxins Serum Levels in Chronic Kidney Disease (Medika2 Study)

**DOI:** 10.3390/jcm10040840

**Published:** 2021-02-18

**Authors:** Maria Teresa Rocchetti, Biagio Raffaele Di Iorio, Mirco Vacca, Carmela Cosola, Stefania Marzocco, Ighli di Bari, Francesco Maria Calabrese, Roberto Ciarcia, Maria De Angelis, Loreto Gesualdo

**Affiliations:** 1 Department of Emergency and Organ Transplantation, Nephrology, Dialysis and Transplantation Unit, “AldoMoro” University, 70124 Bari, Italy; carmela.cosola@uniba.it (C.C.); ighli.dibari.uniba@gmail.com (I.d.B.); loreto.gesualdo@uniba.it (L.G.); 2 Nephrology, AORN San G. Moscati, 83100 Avellino, Italy; br.diiorio@gmail.com; 3 Department of Soil, Plant and Food Science, “Aldo Moro” University, Bari, Via G. Amendola 165/a, 70126 Bari, Italy; mirco.vacca@uniba.it (M.V.); frankof.calabrese@gmail.com (F.M.C.); maria.deangelis@uniba.it (M.D.A.); 4 Department of Pharmacy, University of Salerno, 84084 Fisciano, Italy; smarzocco@unisa.it; 5 Department of Veterinary Medicine and Animal Productions, Faculty of Veterinary, University of Naples, 80138 Naples, Italy; roberto.ciarcia@unina.it

**Keywords:** CKD, intestinal microbiome, ketoanalogs, indoxyl sulfate, p-cresyl sulfate, very low protein diet, mediterranean diet

## Abstract

Nutritional therapy (NT) is a therapeutic option in the conservative treatment of chronic kidney disease (CKD) patients to delay the start of dialysis. The aim of this study was to evaluate the specific effect of ketoanalogs (KA)-supplemented diets for gut microbiota modulation. In a previous study we observed that the Mediterranean diet (MD) and a KA-supplemented very-low-protein diet (VLPD) modulated beneficially gut microbiota, reducing indoxyl- and p-cresyl-sulfate (IS, PCS) serum levels, and ameliorating the intestinal permeability in CKD patients. In the current study, we added a third diet regimen consisting of KA-supplemented MD. Forty-three patients with CKD grades 3B–4 continuing the crossover clinical trial were assigned to six months of KA-supplemented MD (MD + KA). Compared to MD, KA-supplementation in MD + KA determined (i) a decrease of *Clostridiaceae*, *Methanobacteriaceae*, *Prevotellaceae*, and *Lactobacillaceae* while *Bacteroidaceae* and *Lachnospiraceae* increased; (ii) a reduction of total and free IS and PCS compared to a free diet (FD)—more than the MD, but not as effectively as the VLPD. These results further clarify the driving role of urea levels in regulating gut integrity status and demonstrating that the reduction of azotemia produced by KA-supplemented VLPD was more effective than KA-supplemented MD in gut microbiota modulation mainly due to the effect of the drastic reduction of protein intake rather than the effect of KA.

## 1. Introduction

Nutritional therapy (NT) is a key component of care during chronic kidney disease (CKD). It has not been recently applied [1,2] but is still used [3,4], although it has always been debated for a long time. Over the years, NT with a protein intake of 0.6 g/kg bw/day was replaced with an even lower NT protein content (0.3 g/kg bw/day) with the addition of a mixture of essential amino acids (EAA) and ketoanalogs (KA) of non-essential amino acids [4,5] resulting, basically, in a vegetarian diet (very-low-protein diet or VLPD), prescribed in advanced chronic kidney disease to reduce the uremic status [5]. While KA use endogenous urea nitrogen to generate the corresponding amino acid, EAA satisfy the body’s nitrogen need, reducing the risk of nutritional disorders. The supplemented VLPD, is a protein-restricted dietary regimen remarkably influencing protein synthesis, therefore it is often proposed for patients suffering from moderate to advanced chronic kidney disease [6,7]. KA are capable of inducing a great reduction of urea to levels comparable to those of healthy individuals [8], contributing to restore gut microbiome balance. In turn, urea and the uremic milieu are responsible for the alteration of gut microbiota and the increased uremic toxins production in a vicious circle in which both the kidney damage and gut dysbiosis increase [9]. It has become apparent that the microbiota’s metabolites play an important role in the incidence of cardiovascular disease in CKD [10]. In particular, protein-bound uremic toxins such as indoxyl sulfate (IS) and p-cresyl sulfate (PCS), because of their high binding affinity to albumin, cannot be efficiently removed by conventional hemodialysis, causing their progressive accumulation in CKD with the disease progression. It has been widely demonstrated that the IS and PCS accumulation in CKD patients results in organ damage [10]. Therefore, strategies aimed at lowering their intestinal production are highly needed. Supplemented VLPD is a dietetic–nutritional therapy frequently adopted in the conservative treatment of CKD patients, often integrated with pharmacological treatments to maintain an optimal nutritional status and to prevent the complications related to renal insufficiency [3,11]. The final goal of nutritional therapy is to delay the start of dialysis in end stage renal disease (ESRD) patients, improving quality of life and reducing healthcare costs. The Mediterranean diet (MD) and the low protein diet are useful in the early and moderate stages of CKD [12,13,14] and, as well as the supplemented VLPD, they are able to reduce the progression of CKD and cardiovascular risk [3,11,15]. Recently, we demonstrated that nutritional therapy, namely MD and, to a greater extent, the supplemented VLPD, were effective in manipulating the intestinal microbiota and in reducing azotemia and serum levels of protein-bound uremic toxins in CKD patients [16]. The present cross-over randomized controlled trial (RCT)was designed in order to evaluate the specific effect of KA and the role of azotemia on gut microbiota composition, uremic toxins production, and gut barrier permeability. In this regard, it was particularly interesting to study the effects of KA-supplemented MD compared to MD (without KA) and supplemented VLPD.

## 2. Materials and Methods

### 2.1. Study Design

**Scheme 1:**
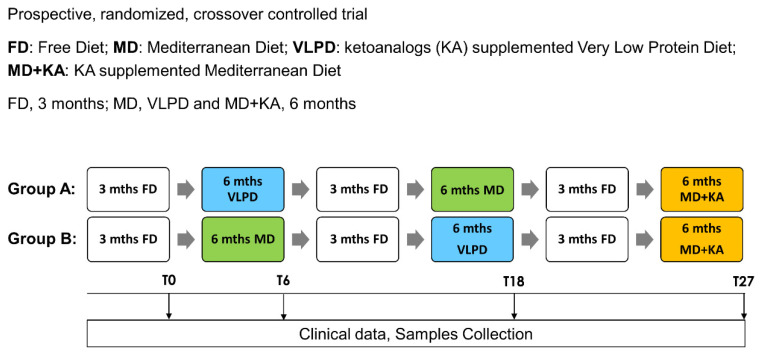
Study Design MEDIKA2.

This study involved 43 incident CKD stage 3b to 4 patients who were willing to continue the prospective, randomized, crossover-controlled trial [16] by undergoing the third arm of the diet, namely MD + KA. The complete study design is reported in Scheme 1. The study was carried out in accord to the Declaration of Helsinki (IV Adaptation) and all participants signed the informed consent at inclusion. The study protocol was registered on ClinicalTrials.gov with the identifier number NCT02302287 and approved by the local institutional Ethical Review Board (Campania Nord Ethics Committee, Avellino, Italy. Code Number: CECN/296). Patients’ recruitment inclusion criteria, detailed diets, primary and secondary end points, medical visits, and NT adherence have been reported in our previous works [8,16]. Software randomization split CKD patients into 2 treatment groups (A, B), with a 1:1 ratio as reported in Scheme 1.

The different dietary regimes (Table 1) have already been fully described previously [8,16], with the exception of the new third arm, MD + KA, which implies the oral administration of one tablet of essential amino acids (285 mg/kg of body weight) and ketoanalogs (323 mg/kg of body weight) (ALFA-KAPPA, Fresenius Kabi) [17] for every 5 kg of the patient’s ideal body weight, provided at breakfast, lunch, and dinner in patients following the Mediterranean diet (Table 1). 

To avoid any potential carry-over effect of the diet regimens (MD, VLPD, and MD + KA) a washout period of free diet (FD) (three months) followed each cross-over timing. Biological samples (sera, stools, and urine) from each patient were collected at the time of recruitment (T0), and after three (T3), nine (T9), twelve (T12), eighteen (T18), twenty-one (T21), and twenty-seven (T27) months; times at which medical examinations were also carried out. 

### 2.2. Collection of Serum and Fecal Samples

Blood and fecal samples were collected and processed as described [16]. 

### 2.3. Reagents

All reagents used have been previously described [16].

### 2.4. LC-MS/MS for Quantification of PCS and IS

Indoxyl sulfate and p-cresyl sulfate serum levels have been analyzed for all 43 CKD patients at the end of three months of FD (wash-out period, baseline) and at the end of each diet regimen. A total of four measures were registered for each patient. Total and free IS and PCS were measured by multiple-reaction-monitoring mass spectrometry analysis as described in our previous work [16,18].

### 2.5. Serum D-Lactate Assay

D-lactate serum levels were analyzed in all 43 CKD patients at the different time points as described [16]. 

### 2.6. RNA Extraction from Fecal Samples and 16S cDNA Sequencing

Total RNA was extracted from all fecal samples, transcribed to cDNA, and analyzed by NGS (Illumina MiSeq desktop sequencer) as described previously [16].

### 2.7. Statistics

No sample size estimation was carried out because no data were present in current literature on the effects of diets. Variables were reported as appropriate: mean ± standard deviation (SD), median and interquartile range (IQR), or count (percentage). Differences occurring among the different dietary regimens were assessed by Student’s t-test or Wilcoxon rank sum test as appropriate. All analyses were conducted as intention-to-treat. Two-tailed probability values < 0.05 were considered statistically significant. Analyses were completed using R version 3.1.3 (2015-03-09) (The R Foundation for Statistical Computing, Vienna, Austria). For linear regression analysis, the Statview software package SAS 5.0 version was used. Data obtained by 16S rRNA gene amplicon sequencing were analyzed by Principal Component Analysis (PCA) based on Unifrac distance metric and taxonomic abundance [19,20] using the statistical software Statistica for Windows (Statistica 6.0 for Windows 1998, StatSoft, Vigonza, Italia). Measures of diversity were screened for group differences by the use of ANOVA. In order to identify clusters among samples (dietary interventions) and variables (bacterial taxa) the Permut-MatrixEN software was also used [21]. Multivariable association between 16S rRNA data abundances at different taxonomic levels and dietary features was performed using the MaAsLin2 R package (https://huttenhower.sph.harvard.edu/maaslin/ (doi.org/10.1101/2021.01.20.427420; accessed on 20 January 2021)). In order to obtain correlation scores between operational taxonomic units (OTUs) and metabolite concentration, a Spearman correlation analysis was applied. The R- analyses were performed using the vegan, labdsv, DESeq2, and phyloseq packages.

## 3. Results

### 3.1. Patients

All CKD patients completed the trial. Table 2 shows the list of demographic, biochemical, and clinical data of all 43 patients in accordance with each nutritional regimen. In comparison with MD, KA-supplemented MD increased diastolic blood pressure, decreased HbA1c, increased triglycerides and transferrin, and decreased creatinine clearance. 

Differently, VLPD lowered both the systolic and diastolic blood pressure, the serum creatinine, urea, sodium, phosphorus, parathyroid hormone, proteinuria, and C reactive protein more effectively than MD or KA-supplemented MD. At serum level, VLPD was associated with increased bicarbonate and eGFR (estimated glomerular filtration rate), while at urine level VLPD was associated with increased potassium and decreased sodium and phosphate (Table 2). Only VLPD lowered the urinary phosphorus excretion and sodium intake according to literature data [8,11,22,23].

### 3.2. Microbial Components Linked to Specific Dietary Intake

**Figure 1:**
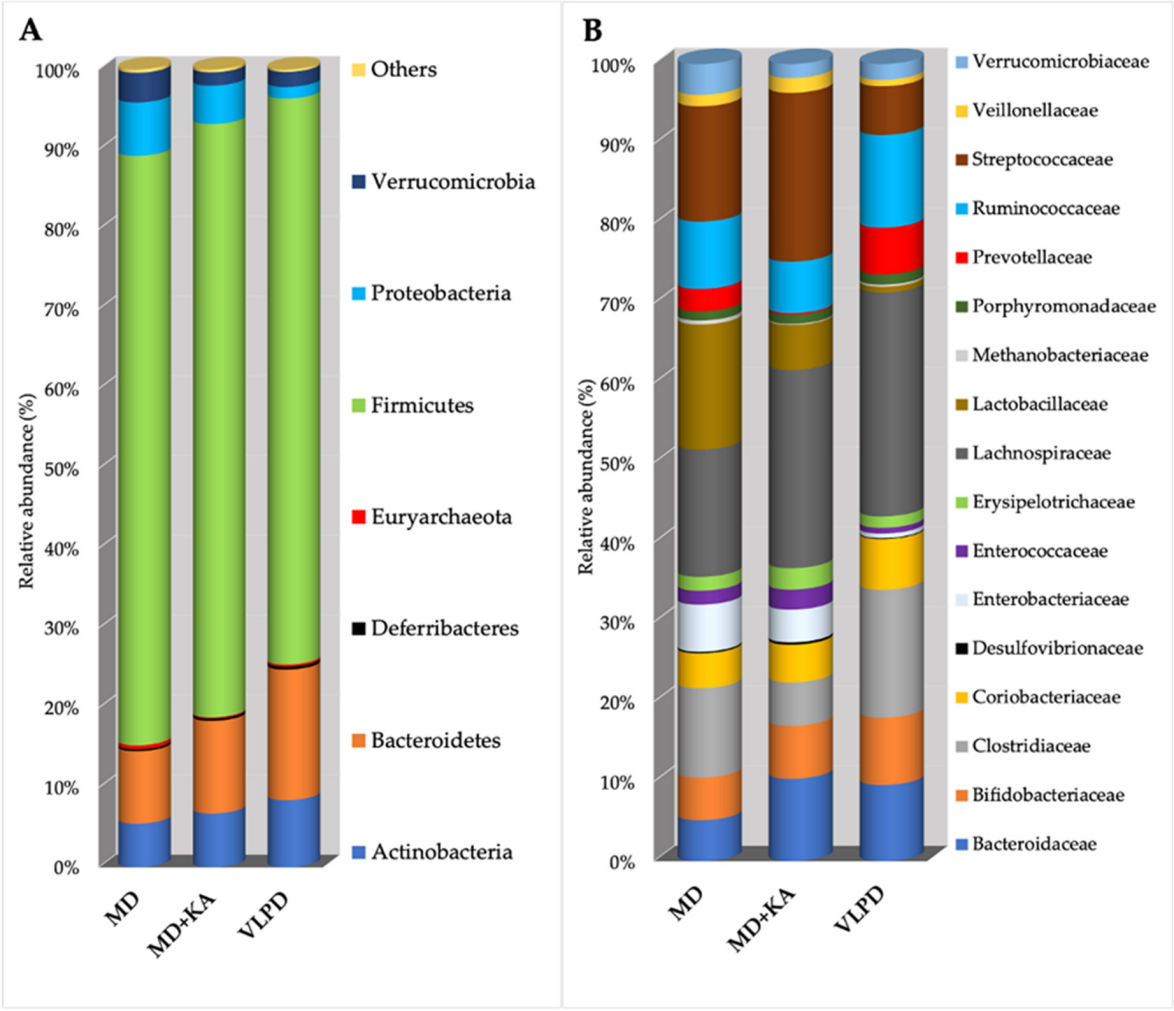
Fecal microbiota composition in chronic kidney disease (CKD) patients associated with different dietary regimens. Three different dietary regimens were evaluated: the Mediterranean diet (MD), Mediterranean diet supplemented with ketoanalogs (MD + KA), and the very-low-protein diet supplemented with ketoanalogs (VLPD). The panel on the left (**A**) shows the relative abundance (as percentual average) of gut bacterial phyla found in CKD patients after each dietary regimen. Phyla with a relative abundance <0.1% in all samples were grouped together and named “Others”. The right panel (**B**) shows the relative abundance (as percentual average) of gut bacterial families (>1% at least in one sample) found in the feces of CKD patients after each dietary regimen.

The microbial alpha diversity values were not affected by the four different dietary regimens (*p* > 0.05). Similarly, there was no strict separation concerning microbiota composition in MD, MD + KA, and VLPD when performed a principal components analysis (PCA). At phylum level, the relative abundance of *Bacteroidetes* was highest after the VLPD (16.37%), significantly when compared to the MD (9.09%; *p* = 0.03) (Figure 1A). Although not significantly, the VLPD determined also an increase of *Actinobacteria* (MD: 5.47%, MD + KA: 6.74%, VLPD: 8.41%) and a decrease of *Firmicutes* (MD: 73.93%, MD + KA: 74.37%, VLPD: 70.93%). The main effect of the VLPD was on *Proteobacteria* abundances, which were strongly reduced compared to other diets (*p* ≤ 0.022). KA supplementation combined with the MD (MD + KA) determined a decrease of the *Euryarchaeota* (*p* = 0.002). 

At family level, the VLPD affected the amounts of *Desulfovibrionaceae* (0.12%), *Enterobacteriaceae* (0.53%), both families of *Proteobacteria*, and *Streptococcaceae* (*Firmicutes*), determining the lowest relative abundances (*p* ≤ 0.039; Figure 1B). Compared to MD + KA, also *Enterococcaceae*, *Erysipelotrichaceae*, and *Veillonellaceae* decreased in the VLPD (*p* ≤ 0.031). Conversely, in the VLPD was found the highest levels of *Ruminococcaceae* (11.13%, *p* ≤ 0.024). Based on the abundances in MD, the KA supplementation in MD + KA seems to decrease levels of *Clostridiaceae*, *Methanobacteriaceae*, and *Prevotellaceae* (*p* ≤ 0.042). Otherwise, this was not confirmed in VLPD where *Clostridiaceae* and *Prevotellaceae* increased (*p* ≤ 0.009). The MD was mainly characterized by high abundances of *Lactobacillaceae* (15.1%), but the KA supplementation determined their decrease in MD + KA (5.39%; not significant) and particularly in the VLPD (0.69%; *p* = 0.01). *Bacteroidaceae* were mainly found in both MD + KA (9.87%) and the VLPD (9.15%) and reduced in the MD (4.95%; *p* = 0.006). The taxon that seems to report a direct relationship with KA supplementation was *Lachnospiraceae*. This family reported an increased abundance in both the supplemented dietary regimens (MD + KA and VPLD), even if the increase was significantly only comparing the MD to the VLPD (*p* ≤ 0.01).

**Figure 2:**
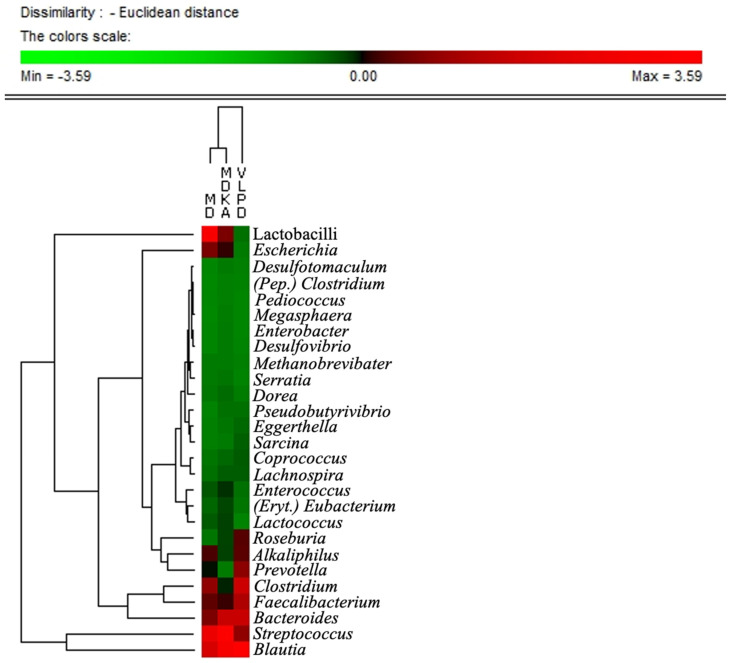
Fecal microbial genera found in chronic kidney disease (CKD) patients associated with different diets: Mediterranean diet (MD), ketoanalog-supplemented Mediterranean diet (MD + KA), and ketoanalog-supplemented very-low-protein diet (VLPD). Only statistical different (*p* < 0.05) genera at least in one dietary regimen were reported. Eryt. -*Eubacterium*: genus *Eubacterium* belongs to the *Erythrobacteraceae* family; Pep. -*Clostridium*: genus *Clostridium* belongs to the *Peptostreptococcaceae* family.

The *Lachnospiraceae* modulation associated with the KA supplementation was also observed at genus and species levels; in this line, *Blautia* (*Bl. coccoides*), *Lachnospira* (*L. pectinoschiza*), and *Roseburia* (*R. faecis*) were taxa that reported a marked increase in both KA-supplemented dietary regimens in comparison with the MD (*p* ≤ 0.029; Figure 2, Table 3). Meanwhile, other OTUs belonging to *Lachnospiraceae* were mainly harbored by the dietary change in the VLPD, e.g., *Bl. hydrogenotrophica*, *Bl. obeum*, *Bl. wexlerae*, *Coprococcus eutactus*, and *Pseudobutyrivibrio xylanivorans* (Table 3). Oppositely, *Dorea* (*D. formicigenerans*) was the only taxon of this family that decreased in the VLPD compared to both the MD and MD + KA (*p* ≤ 0.005). The VLPD confirmed the different effect on firmicutes sub-taxa; this dietary regimen mainly decreased lactobacilli abundances (*L. fermentum*, *L. gasseri*, *L. oris*, *L. salivarius; p* ≤ 0.045) and *Streptococcus* (*S. parasanguinis*, *S. sobrinus*, and *S. vestibularis; p* ≤ 0.043). Moreover, the VLPD also significantly decreased abundances of *Enterococcus lactis* and *Eubacterium biforme* compared to the MD + KA (*p* ≤ 0.024).

Conversely, *Firmicutes* OTUs harbored by the low protein intake were: *Clostridium cadaveris* and *Sarcina maxima* (both *Clostridiaceae*; *p* ≤ 0.021), *Eggerthella sinensis* (*p* ≤ 0.002), and *Faecalibacterium prausnitzii* (*p* ≤ 0.007). Among *Bacteroidetes*, only *Bacteroides stercoris* was strictly influenced by the VLPD, increasing compared to the MD and MD + KA (*p* ≤ 0.019). Undoubtedly, the VLPD decreased proteobacteria OTUs (in particular *Escherichia albertii* and *Serratia entomophila*; *p* ≤ 0.033) and increased *Bifidobacteriaceae* abundances (*B. adolescentis* and *B. stercoris*; *p* ≤ 0.038) compared to both the other dietary regimes (the MD and MD + KA).

### 3.3. Microbial Pattern Linked to Ketoanalogs Administration

**Figure 3:**
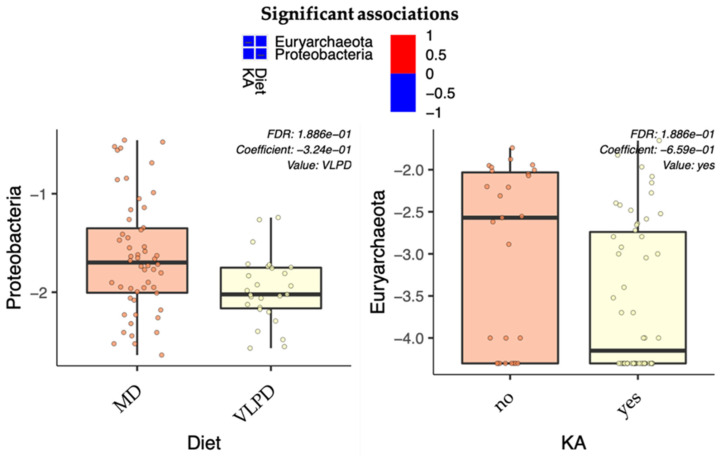
Multivariable associations between phylum abundances and dietary features. The MD-boxplot includes both Mediterranean diets with (MD + KA) and without (MD) ketoanalog supplementation. Both diets supplemented with ketoanalogs (Mediterranean diet (MD + KA) and the very-low-protein diet (VLPD)) are included in the “yes” KA boxplot.

In order to determine microbial patterns mainly influenced by the different diets (MD or VLPD) and which ones were influenced by the ketoanalog administration, we performed a statistical analysis in MaAsLin2. We analyzed all bacterial abundances based on two associated metadata used as fixed effects: diet, MD (used for both MD and MD + KA) or VLPD; and ketoanalogs either administered (MD + KA and VLPD) or not administered. At phylum level (Figure 3), it was observed that proteobacteria were negatively associated with the VLPD (*p* = 0.023), whereas *Euryarchaeota* reported a negative association with KA consumption (*p* = 0.012). However, both the afore-reported associations were not confirmed for adjusted *p*-values (q-value > 0.05; Figure 3). Meanwhile, at family level (Table 4) was found a negative association of *Streptococcaceae* and *Lactobacillaceae* with the VLPD (*p* ≤ 0.000; q ≤ 0.016), a positive association of *Clostridiaceae* with the VLPD (*p* < 0.000; q = 0.015), and a positive correlation between *Lachnospiraceae* and KA consumption (*p* = 0.002; q = 0.042). Similar results were found also at genus level (Table 4), where *Streptococcus*, lactobacilli, and *Clostridium* reported the same trend of the relative families (*p* < 0.000; q ≤ 0.02). Meanwhile, despite the positive correlation between *Lachnospiraceae* and KA consumption, *Dorea* reported a negative correlation based on the VLPD dietary regimen (*p* < 0.000; q = 0.002).

### 3.4. Uremic Toxins

**Figure 4:**
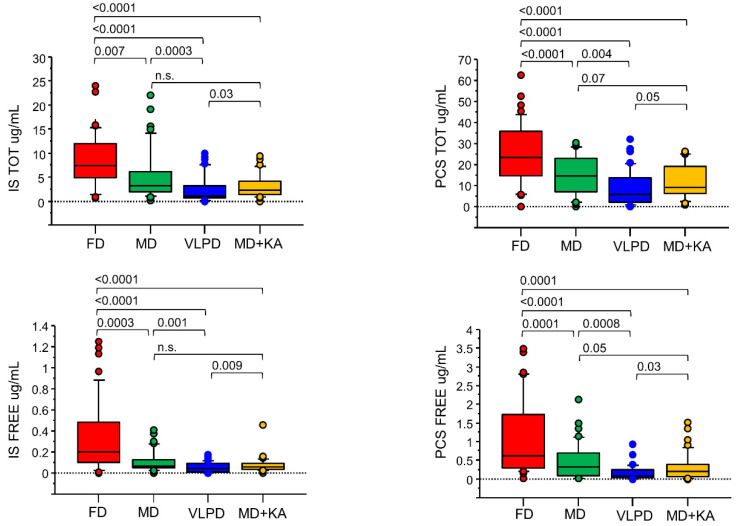
Serum levels of IS and PCS measured by multiple-reaction-monitoring mass spectrometry analysis. Serum levels of IS and PCS in CKD patients in accordance with the different diets (Table 1). *p* values were calculated by the Wilcoxon test.

In comparison to FD (IS: 8.65 ± 6.0 μg/mL; 0.3 ± 0.3 μg/mL. PCS: 25.2 ± 14.4 μg/mL; 1.0 ± 0.98 μg/mL) the VLPD was able to reduced total and free IS (2.5 ± 2.8 μg/mL, *p* < 0.0001; 0.05 ± 0.05 μg/mL, *p* < 0.0001) and PCS (8.9 ± 8.1 μg/mL, *p* < 0.0001; 0.2 ± 0.2 μg/mL, *p* < 0.0001) serum levels more effectively than other dietary regimens. The MD also decreased the levels of total and free IS (5.3 ± 5.6 μg/mL, *p* = 0.007; 0.15 ± 0.3 μg/mL, *p* = 0.003) and PCS (14.9 ± 9.5 μg/mL, *p* < 0.0001; 0.54 ± 0.6 μg/mL, *p* = 0.0001) as compared to FD, although not as effectively as the VLPD (Figure 4). 

Likewise, MD + KA reduced the serum levels of total and free IS (2.9 ± 2.3 μg/mL, *p* < 0.0001; 0.07 ± 0.07 μg/mL, *p* < 0.0001) and PCS (11.8 ± 7.9 μg/mL, *p* < 0.0001; 0.31 ± 0.35 μg/mL, *p* = 0.0001) compared to FD—more than the MD, but not as effectively as the VLPD. The total and free IS and PCS serum levels of CKD patients after the MD + KA were not significantly lower than those after the MD regimen.

We found a positive correlation between both total and free IS and PCS serum levels with azotemia (r = 0.38, *p* < 0.0001; r = 0.38, *p* < 0.0001 for total and free IS; r = 0.42, *p* < 0.0001; r = 0.41, *p* < 0.0001 for total and free PCS). In addition, both total and free IS and PCS serum levels showed a positive correlation with protein intake (r = 0.30, *p* = 0,0002, and r = 0.26, *p* = 0.001 for total and free IS, respectively; r = 0.40, and r = 0.40, *p* < 0.0001 for total and free PCS, respectively) and parathyroid hormone (PTH) (r = 0.30, *p* < 0.0001; r = 0.15, *p* =0.05 for total and free IS, respectively; r = 0.32, *p* < 0.0001 and r = 0.27, *p* = 0.0006, for total and free PCS respectively), while only total IS and PCS were weakly negatively correlated with the eGFR values (r = −0.19, *p* = 0.01; r = −0.17, *p* = 0.02) of the enrolled patients. 

### 3.5. KA Supplementation Does Not Reduce Intestinal Permeability Compared to the MD 

**Figure 5:**
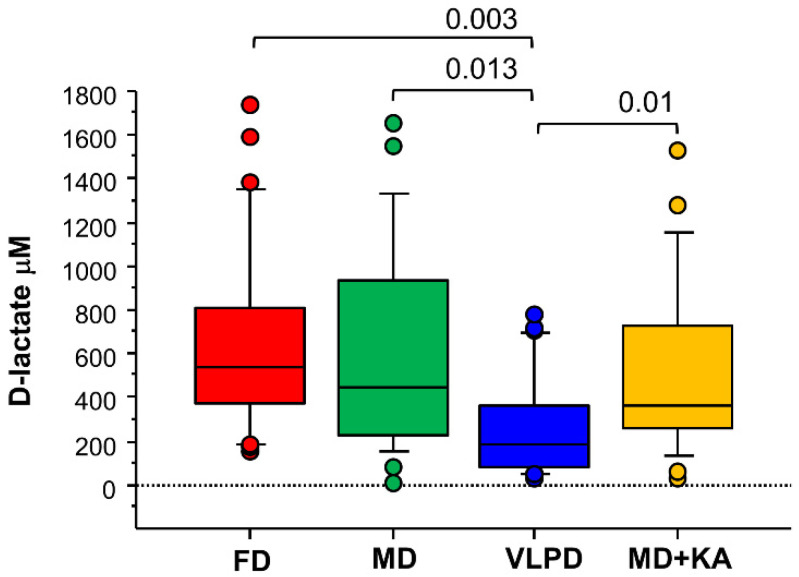
D-lactate serum levels measured in CKD patients in accordance with the different diets (Wilcoxon test).

As a marker of intestinal permeability, serum D-lactate levels were measured in CKD patients following the four different dietary regimens (FD, MD, VLPD, and MD + KA). The VLPD is very effective in reducing D-lactate in CKD patients (266.6 uM), in comparison with FD [651.89 uM, *p* = 0.003] and the MD [590.8 uM, *p* = 0.01], confirming our previous data [16]. The MD regimen showed a not-significant decrease of D-lactate levels in comparison with FD and KA supplementation does not further reduce them (MD + KA, 533.8.8 uM) (Figure 5). We found that D-lactate levels were positively correlated with blood urea nitrogen levels (r = 0.3, *p* = 0.002). 

### 3.6. Correlations between Dietary Intake and Metabolome with Microbiome 

There was a positive relationship between fecal microbiota and azotemia (Figure S1). Lactobacilli and *Streptococcus* were positively correlated (r = 0.44, FDR = 0.02; r = 0.40, FDR = 0.03, respectively) with azotemia. *Streptococcus* was also positively associated with the urinary phosphorus excretion (FeP) (r = 0.41, FDR = 0.03). *Faecalibacterium* was negatively associated with systolic blood pressure (r = −0.49, FDR = 0.03). Among protein-bound uremic toxins, free IS was found to be negatively associated with *Roseburia* (r = −0.49, FDR = 0.03) (Figure S1).

## 4. Discussion

As we have recently proved [16], nutritional therapy represents an effective strategy to modify the gut microbiome and to lower the serum levels of protein-bound uremic toxins in CKD patients. Indeed, the Mediterranean diet and, to a greater extent, the VLPD have proven to be effective in beneficially modulating gut microbiota, reducing IS and PCS serum levels, and intestinal permeability in CKD patients [16]. The aim of the current trial was to investigate the effects of the ketoanalogs (KA) on microbiome modulation, uremic toxins levels, and intestinal permeability. KA are precursors of the corresponding amino acids, in which they are converted by transamination, a chemical reaction that transfers an amino group from urea to a ketoacid to form the amino acid. This reaction allows the consumption of the available nitrogen in excess, a condition often occurring in chronic kidney impairment, consequently lowering urea concentration [1]. Moreover, in order to maintain a good nutritional status despite the reduced protein intake that is a feature of the VLPD, essential amino acids (EAAs) are normally administered together with KA. Literature data indicate that the VLPD supplemented with KA prevents hyperparathyroidism, insulin resistance, and accumulation of uremic retention solutes— ameliorating renal function and nutritional status [6,16,22,24,25,26,27,28]. Whether isolated supplementation of KA, not associated with low-protein diets, has any benefit on metabolic alterations related to CKD, remains still unexplored. Milovanova and coworkers published a comparative study of LPD supplemented with KA and LPD alone in CKD 3b–4 stage patients in relation to serum klotho and FGF-23 levels, key markers of cardiovascular complications and CKD progression. They found lower FGF-23, and higher Klotho in the LPD + KA group compared to the LPD one, concluding that KA supported the nutritional status and was able to correct FGF-23 and Klotho abnormalities involved in cardiovascular calcification and cardiac remodeling decrease in CKD [24]. Another study on obese CKD patients showed that supplementation of KA in long-term administration of a LPD contributed to lower ADMA (asymmetric dimethylarginine), visceral body fat, and proteinuria compared to LPD alone. In the group with co-administration of KA and LPD there was also a decrease of glycated hemoglobin, LDL-cholesterol, and pentosidine, which potentially contributed to slow down the progression of kidney failure [29]. The same group designed a long-term, prospective study on patients with chronic renal failure subjected to three therapeutic protocols: LPD plus KA plus erythropoietin (EPO), LPD plus EPO, and LPD alone [30]. In the first group, after three years’ follow up, serum urea, proteinuria, total cholesterol, LDL-cholesterol levels, and plasma triglyceride levels declined, whereas albumin and HDL-cholesterol levels increased compared to groups LPD plus EPO and LPD alone [30]. These data partially support our findings such as the significant reduction of glycated hemoglobin after MD + KA regimen compared to the MD alone and the VLPD, as well as the tendency to decrease of proteinuria after the MD + KA regimen compared to the MD alone. On the contrary, KA supplementation to the MD increased triglyceride levels compared both the MD alone and the VLPD. This apparent discrepancy with the cited paper could be attributed to EPO effects on the carbohydrate metabolism, which improvement is associated with decreased serum triglycerides [31]. Furthermore, supplementation of KA to MD did not increase the albumin and cholesterol in our patients, as reported in previous investigations [29,30].

The gut microbiota involvement in dietary metabolism is widely recognized [8,32,33]. A strict relationship between gut microbial patterns and different dietary regimen has been demonstrated [16,34]. Similarly, in the present study many of the detected changes were observed strictly related to the VLPD more than arising from KA supplementation in the MD. First of all, a decrease in *Proteobacteria* abundances was found in the VLPD. Although KA supplementation in MD + KA determined a lesser amount of free nitrogenous metabolites into intestinal lumen, this seems not sufficient to reduce the bioavailability of elective substrates for *Proteobacteria* proliferation. As is well-known, a significant decrease of *Proteobacteria* in the intestinal milieu of CKD patients represents one of the main outcomes expected from nutritional therapies [35]. *Proteobacteria*, indeed, play a pivotal role in metabolizing urea and ammonium into uremic toxins [36,37], and higher proteobacteria subtaxa, in particular *Enterobacteriaceae* and *E. coli.*, have been associated with an injured functionality of kidneys [38]. However, the observed decrease of *Euryarchaeota* associated with KA supplementation (in both MD + KA and VLPD) underlined the effectiveness of KA in reducing, but not significantly, the nitrogenous metabolites such as urea and ammonium. In this line, high presence of *Euryarchaeota* and relative subtaxa was previously associated with high urea and NH_4_ bioavailability [39]. For this reason and considering also their direct contribution to the trimethylamine-(TMA) metabolism, taxa of *Euryarchaeota* were previously associated with different diseases [40]. On the other side, in our study MD + KA reported a trend in reduction of *Lactobacillaceae* abundances, finding confirmed by the significant negative relationship with the VLPD at both family and genus level. The proteolytic metabolism of lactobacilli towards different substrates (e.g., milk and derivates, meat, gluten) has been widely reported [41,42,43] and probably due to this we detected a positive relationship between lactobacilli and azotemia. Otherwise, *Lactobacillaceae* and lactobacilli repeatedly showed beneficial contributes towards the host health [44]. Postbiotic metabolites of lactobacilli directly impact on gut health [45] as well as suppressing the expansion of potential pathogenic microorganisms [46,47]. Moreover, they are able to produce short chain fatty acids (SCFAs), i.e., acetic, propionic, and butyric acids [48]. Acetate and butyrate are essential in CKD patients, in which the accumulation of urea and ammonia drives the pH out of normal range [49], whereas SCFAs could bring back pH ranges into normal ones [50]. Additionally, lactobacilli and derived SCFAs contributes also to enhance the epithelial layer integrity [51,52,53], therefore reducing the translocation of microbes and metabolites (including urea, ammonia, and uremic toxins) into the systemic circulation, an evidence common in CKD patients [54,55]. In the VLPD, the lack in *Lactobacillaceae* was replaced by a significant increase in bifidobacteria, which, at the same of *Lactobacillaceae*, are able to produce SCFAs reporting beneficial contributes to the host health and gut permeability [56]. In the present work, this was confirmed by the significant decrease in gut permeability detected in the VLPD. The same was not observed in MD + KA in which both lactobacilli and bifidobacteria were reduced compared to the MD and VLPD, respectively. Herein, we observed an increase of *Clostridiaceae* and *Clostridium* (*sensu stricto*) positively associated with the VLPD regimen. On note, the observed clostridial increase in the VLPD was mainly related to abundances of *Clostridium cadaveris*, an OTU that recently has been associated with the indole-propionic acid (IPA) metabolism [57]. This was interestingly considering that it could result from the strictly vegetable-based diet (i.e., VLPD) as previously reported by Tuomainen et al. [58], and it was surprisingly considering that in other clinical trials performed on CKD patient IPA was considered as a healthy biomarker [59]. In the same line, the high adherence to the VLPD probably determined the detected increase of glycated hemoglobin and triglyceride levels resulting from the polysaccharides digestion due to the *Lachnospiraceae* metabolism. *Blautia* and *Roseburia*, both genera of *Lachnospiraceae*, were found increased in both KA-supplemented diets. Taking into account that *Lachnospiraceae* are butyrogenic bacteria, this bacterial family reported a controversial role in health and disease [60]. *Lachnospiraceae* were reported as the main producers of SCFAs into the gut environment; otherwise, they harvest a great amount energy from diet metabolizing carbohydrates, as markedly codifying butyril-CoA:acetate CoA and butyrate kinase [60]. Additionally, considering the ability of different species of *Blautia*, in particular *B. obeum* and *B. hydrogenotrophica*, to metabolize tyrosine and tryptophan into *p*-cresol, phenol, and indole [61,62] their elevated abundances into the intestinal lumen of CKD patients cannot be considered as a positive outcome.

Overall, the KA-supplemented MD was not more effective than the MD, or as effective as the VLPD, in lowering serum levels of total and free IS and PCS and in improving the intestinal permeability, although the serum levels of both uremic toxins and of D-lactate tended to be lower after the MD + KA regimen compared to the MD alone. These data suggest that the KA-supplemented MD could still be beneficial in those patients with CKD who fail to undergo a very restrictive protein diet such as the VLPD.

In parallel, the reduction of the urea load caused by the transamination of KA into the corresponding amino acids is not sufficient to exert a beneficial modulation (saccharolytic shift) of the microbiota when not accompanied by an overall restriction of protein intake, as in the VLPD. To this purpose, the results arising from the present study further clarify the driving role of azotemia in regulating gut integrity status. Gut permeability ameliorates when urea levels decrease, like after a VLPD but not after the MD supplemented with KA. Rather, KA seem to act more at metabolic level on glycemic status, lipid levels, and iron pattern, as evidenced in MD + KA group by amelioration of HbA1c and transferrin levels and by increase of serum triglycerides, the latter probably related to gut microbiota metabolism. We argue that further study, with extended follow up periods and with a larger number of uremic patients are needed to evaluate and to compare the efficacy of these NT on clinical outcomes, such as the start of dialysis, *in primis*, and the worsening residual renal function. Finally, we can conclude that in the supplemented VLPD, both KA and the very-low-protein intake act synergistically in the modulation of microbiota and in the consequent amelioration of protein-bound uremic toxins (PBUTs) levels and gut permeability.

## Figures and Tables

**Table 1:** Diets’ composition [8,16].

	Mediterranean Diet	Very-Low-Protein Diet + KA	Mediterranean Diet + KA
Protein Intake, (g/kg bw/day)	0.8	0.3	0.8
Animal Protein (g/day)	30–40	zero	30–40
Vegetal Protein (g/day)	40–50	30–40	30–40
Total Fat (Saturated)	34% (4%)	32% (4%)	34% (4%)
Carbohydrates	58%	62%	58%
Sugar	11%	11%	11%
Fibres (g/day)	20	22	20
Energy (kcal/kg bw/day)	30–35	30–35	30–35
Sodium (g/day)	5–6	5–6	5–6
Potassium (g/day)	2–4	3–5	3–5
Calcium (g/day)	1.1–1.3	1.1–1.3	1.1–1.2
Phosphorus (g/day)	1.2–1.5	0.6–0.8	1.0–1.2
Fe (mg/day)	9	5	9
Ketoanalogs (cpr/5 kg bw/day)	zero	1	1

**Table 2:** Anthropometric, biochemical, clinical data of patients according to each nutritional regimen.

	Free Diet	Mediterranean Diet–MD	VLPD + KA	MD + KA	*p* Value
Number	43				
Sex (M), number	32				
Age, years	65 ± 15				
Diabetic, n (%)	18 (41.8)				
		
Weight, kg	73 ± 14	73 ± 14	72 ± 13	72 ± 13	n.s
SBP, mm Body	137 ± 20	136 ± 16	127 ± 13 *	134 ± 15	<0.001
DBP, mm Hg	77 ± 12	77 ± 7	73 ± 6 ^§^	80 ± 7 **	0.02
Creatinine, mg/dL	3.6 ± 1.8	3.5 ± 1.4	3.2 ± 1.3 *	3.5 ± 1.5	0.006
eGFR, ml/min/1.73 m^2^	20 ± 11	20.2 ± 10	22.3 ± 12.6 *	20.5 ± 11.2	0.0001
Urea, mg/dL	172 ± 23	133 ± 30 ^#^	62 ± 20 *	130 ± 31 ^#^	<0.001
Glycaemia, mg/dL	118 ± 32	115 ± 30	112 ± 21	113 ± 23	n.s.
Glycated HB (%)	7.4 ± 1.1	7.4 ± 1.0	7.2 ± 0.8	7.1 ± 0.8 ^#^	0.05
Uricemia, mg/dL	6.1 ± 2.3	5.8 ± 1.5	5.0 ± 1.1 ^$^	5.2 ± 1.0 ^$^	0.03
Natrium, mmol/L	140 ± 2	141 ± 2	139 ± 2 ^$^	140 ± 2	0.001
Cloro, mmol/L	104 ± 6	102 ± 5	100 ± 5	103 ± 5	n.s.
Potassium, mmol/L	4.9 ± 0.6	4.8 ± 0.5	4.9 ± 0.5	4.8 ± 0.4	n.s.
Calcium, mmol/L	9.1 ± 0.6	9.3 ± 0.5	9.2 ± 0.4	9.4 ± 0.4	n.s.
Phosphate, mg/dl	4.7 ± 1.0	4.3 ± 0.8 ^#^	3.7 ± 0.5 *	4.3 ± 0.6 °	<0.001
Bicarbonates, mmol/L	21 ± 3	23 ± 3 ^#^	25 ± 2 *	23.6 ± 2 °	0.001
Cholesterol, mg/dL	166 ± 39	163 ± 37	166 ± 37	167 ± 29	n.s.
Triglycerides, mg/dL	143 ± 78	147 ± 37	149 ± 62	167 ± 29 ^£^	0.03
Sideremia	80 ± 33	71 ± 24	80 ± 26 ^£^	82 ± 32	0.02
Trasferrin	214 ± 42	211 ± 46	201 ± 46	220 ± 52 ^δ^	0.01
Ferritin, ng/ml	135 ± 93	155 ± 295 ^#^	111 ± 68	116 ± 83	0.03
PTH, pg/ml	239 ± 170	241 ± 199	170 ± 87 ^$^	215 ± 177	0.001
Haemoglobin, g/dL	11.6 ± 1.8	11.8 ± 1.5	12.0 ± 1.3	11.8 ± 1.8	n.s.
Albumin, g/dL	3.7 ± 0.5	3.8 ± 0.4	3.8 ± 0.3	3.8 ± 0.4	n.s.
CRP, mg/L	7.0 ± 12	4.3 ± 4.8	2.4 ± 2.0 *	4.3 ± 6.2 °	0.01
Diuresis, ml/day	2120 ± 410	2086 ± 511	2028 ± 359	2124 ± 392	n.s.
Urinary natrium, mmol/day	163 ± 47	137 ± 36	122 ± 28 *	144 ± 50	0.001
Urinary potassium, mmol/day	43 ± 13	47 ± 14 ^#^	55 ± 14 *	49 ± 16 °	0.001
Urinary phosphate, mmol/day	679 ± 150	521 ± 174 ^#^	304 ± 104 *	490 ± 178	0.001
Urinary Cl, mmol/day	130 ± 43	114 ± 26	103 ± 17 *	121 ± 40	<0.001
Prot-u, mg/day	1156 ± 1263	1083 ± 980	936 ± 814 ^$^	1035 ± 964	0.02
Creatinine clearance, mL/min	24 ± 14.3	22.6 ± 13.0	23.6 ± 12.8 ^§^	20.8 ± 12.5 ^£^	0.003
P intake, mg/day	1013 ± 224	797 ± 242 ^#^	484 ± 141 *	746 ± 263	<0.001
Na intake, g/day	9.6 ± 1.9	8.0 ± 1.6	6.8 ± 1.4 *	8.8 ± 2.6	<0.001
Protein intake	1.14 ± 0.2	0.96 ± 0.15 °	0.49 ± 0.12 *	1.0 ± 0.2 °	<0.001

SBP: Systolic blood pressure; DBP: diastolic blood pressure; PTH: parathyroid hormone; UUN: urinary urea nitrogen; CRP: C-Reactive Protein. * *p* < 0.05 vs. FD, MD and MD + KA. ** *p* < 0.05 vs. FD, MD and VLPD. ^#^ *p* < 0.05 vs. FD. ^£^ *p* < 0.05 vs. MD. ^δ^ *p* < 0.05 vs. VLPD. ^$^ *p* < 0.05 vs. FD and MD. ° *p* < 0.05 vs. FD e VLPD. ^§^ *p* < 0.05 vs. MD and MD + KA. * Bonferroni test: *p* < 0.05 vs. FD and MD. ^#^ Bonferroni test: *p* < 0.05 vs. FD.

**Table 3:** Dietary changes in gut microbiota composition of patients with chronic kidney disease (CKD). Significant (*p* < 0.05) bacterial species with a mean of relative abundance (16S rRNA gene amplicon) >0.5% at least in one dietary arm found in feces of CKD patients after three different dietary regimens: the Mediterranean diet (MD), the ketoanalog-supplemented Mediterranean (MD + KA), and the ketoanalog-supplemented very-low-protein diet (VLPD).

Phylum	Family	Species	MD	MD + KA	VLPD	* *p* MD vs. MD + KA	*p* MD vs. VLPD	*p* MD + KA vs. VLPD
Actinobacteria	*Bifidobacteriaceae*	*Bifidobacterium adolescentis*	1.25	1.01	3.39	** ns	0.038	0.022
		*Bifidobacterium stercoris*	0.91	0.92	1.75	ns	0.036	0.014
Bacteroidetes	*Bacteroidaceae*	*Bacteroides eggerthii*	0.2	1.06	0.01	ns	ns	0.024
		*Bacteroides stercoris*	0.74	0.7	1.73	ns	0.019	0.004
		*Bacteroides uniformis*	0.31	1.14	0.84	0.030	ns	ns
	*Prevotellaceae*	*Prevotella copri*	2.51	0.06	5.34	0.036	ns	0.010
Firmicutes	*Clostridiaceae*	*Alkaliphilus crotonatoxidans*	0.91	0.37	0.71	0.028	ns	ns
		*Clostridium cadaveris*	2.23	0.37	7.17	ns	0.019	0.000
		*Sarcina maxima*	0.24	0.18	1.23	ns	0.021	0.014
	*Coriobacteriaceae*	*Eggerthella sinensis*	0.3	0.23	0.89	ns	0.001	0.002
	*Enterococcaceae*	*Enterococcus lactis*	1.31	1.7	0.19	ns	ns	0.024
	*Erysipelotrichaceae*	*Erysipelothrix inopinata*	0.3	0.4	0.57	ns	0.047	ns
		*Eubacterium biforme*	1.12	1.84	0.46	ns	ns	0.008
	*Lachnospiraceae*	*Blautia coccoides*	3.71	6.52	6.59	0.001	0.013	ns
		*Blautia hydrogenotrophica*	0.01	0.27	0.55	ns	0.027	ns
		*Blautia obeum*	1.29	1.24	2.79	ns	0.008	0.001
		*Blautia wexlerae*	2.92	6.62	6.39	ns	0.021	ns
		*Coprococcus eutactus*	0.28	0.62	0.98	ns	0.004	ns
		*Dorea formicigenerans*	0.63	0.71	0.28	ns	0.005	0.001
		*Lachnospira pectinoschiza*	0.83	1.26	1.35	0.020	0.021	ns
		*Pseudobutyrivibrio xylanivorans*	0.24	0.56	0.71	ns	0.036	ns
		*Roseburia faecis*	0.68	1.95	3.87	0.029	0.007	0.022
	*Lactobacillaceae*	*Limosilactobacillus fermentum*	0.6	0.4	0	ns	0.001	0.005
		*Lactobacillus gasseri*	0.73	0.49	0.1	ns	0.045	ns
		*Limosilactobacillus oris*	0.49	0.5	0.03	ns	0.023	ns
		*Ligilactobacillus salivarius*	8.91	1.91	0.11	ns	0.041	ns
	*Ruminococcaceae*	*Faecalibacterium prausnitzii*	3.99	3.38	7.3	ns	0.007	0.001
	*Streptococcaceae*	*Streptococcus parasanguinis*	0.64	1.06	0.05	ns	0.002	0.000
		*Streptococcus sobrinus*	0.78	0.04	0	ns	0.043	ns
		*Streptococcus vestibularis*	7.56	11.89	2.62	ns	0.043	0.000
Proteobacteria	*Enterobacteriaceae*	*Escherichia albertii*	4.85	3.18	0.4	ns	0.029	0.033
		*Serratia entomophila*	0.52	0.38	0.05	ns	0.022	0.022

* *p*: *p*-value. ** ns: not significant (*p* > 0.05).

**Table 4:** Multivariable and significant associations (*p* < 0.05 and q < 0.05) between bacterial abundances (16rDNA gene amplicon sequences) and dietary features. Both Mediterranean diets (MD and MD + KA) were grouped together and compared with the VLPD. Both diets supplemented with ketoanalogs (MD + KA and VLPD) were grouped together in “yes” and compared with the unsupplemented MD (“no”). The column “value” describes to which feature are related the data reported into the same row.

	Metadata	Taxon	Value	Coef	Stderr	Pval	Qval
Family	Diet	*Streptococcaceae*	VLPD	−0.93	0.16	6.04 × 10^−8^	3.62 × 10^−6^
	Diet	*Clostridiaceae*	VLPD	0.38	0.11	5.18 × 10^−4^	1.55 × 10^−2^
	Diet	*Lactobacillaceae*	VLPD	−0.78	0.22	8.21 × 10^−4^	1.64 × 10^−2^
	KA	*Lachnospiraceae*	yes	0.22	0.07	2.85 × 10^−3^	4.27 × 10^−2^
Genus	Diet	*Streptococcus*	VLPD	−0.89	0.16	2.43 × 10^−7^	2.28 × 10^−5^
	Diet	*Dorea*	VLPD	−0.48	0.11	5.92 × 10^−5^	2.78 × 10^−3^
	Diet	*Clostridium*	VLPD	0.47	0.12	2.08 × 10^−4^	6.51 × 10^−3^
	Diet	*lactobacilli*	VLPD	−0.79	0.23	8.83 × 10^−4^	2.08 × 10^−2^

**Abbreviations:** KA, ketoanalogs; MD, Mediterranean diet; MD + KA, Mediterranean diet supplemented with ketoanalogs; VLPD, very-low-protein diet supplemented with ketoanalogs; coef, coefficient of correlation; stderr, standard error; pval, *p*-value; qval, q-value.

## Supplementary Materials

The following are available online at https://www.mdpi.com/2077-0383/10/4/840/s1, Figure S1: Correlations between bacterial genera, metabolome, and dietary intake of CKD patients.
