# Supplementary material for: Ketoanalogs’ Effects on Intestinal Microbiota Modulation and Uremic Toxins Serum Levels in Chronic Kidney Disease (Medika2 Study)
Source: J Clin Med. 2021 Feb 18;10(4):840. doi: 10.3390/jcm10040840 (PMC7922022; doi:10.3390/jcm10040840)

Supplementary Materials

Figure S1. Correlations between bacterial genera, metabolome and dietary intake of CKD patients. The colors of the scale bar denote the nature of the correlation, with 1 indicating a perfectly positive correlation (blue) and -1 indicating a perfectly negative correlation (red) between genus, metabolites and dietary intake. Only significant correlations (FDR, 0.05) are shown. SBP: Systolic blood pressure; DBP: diastolic blood pressure; Na, K, Cl, Ca P: sodium, potassium, chlorine, calcium and phosphorus serum levels; FeNa: urinary sodium excretion; FeP: urinary phosphorus excretion.

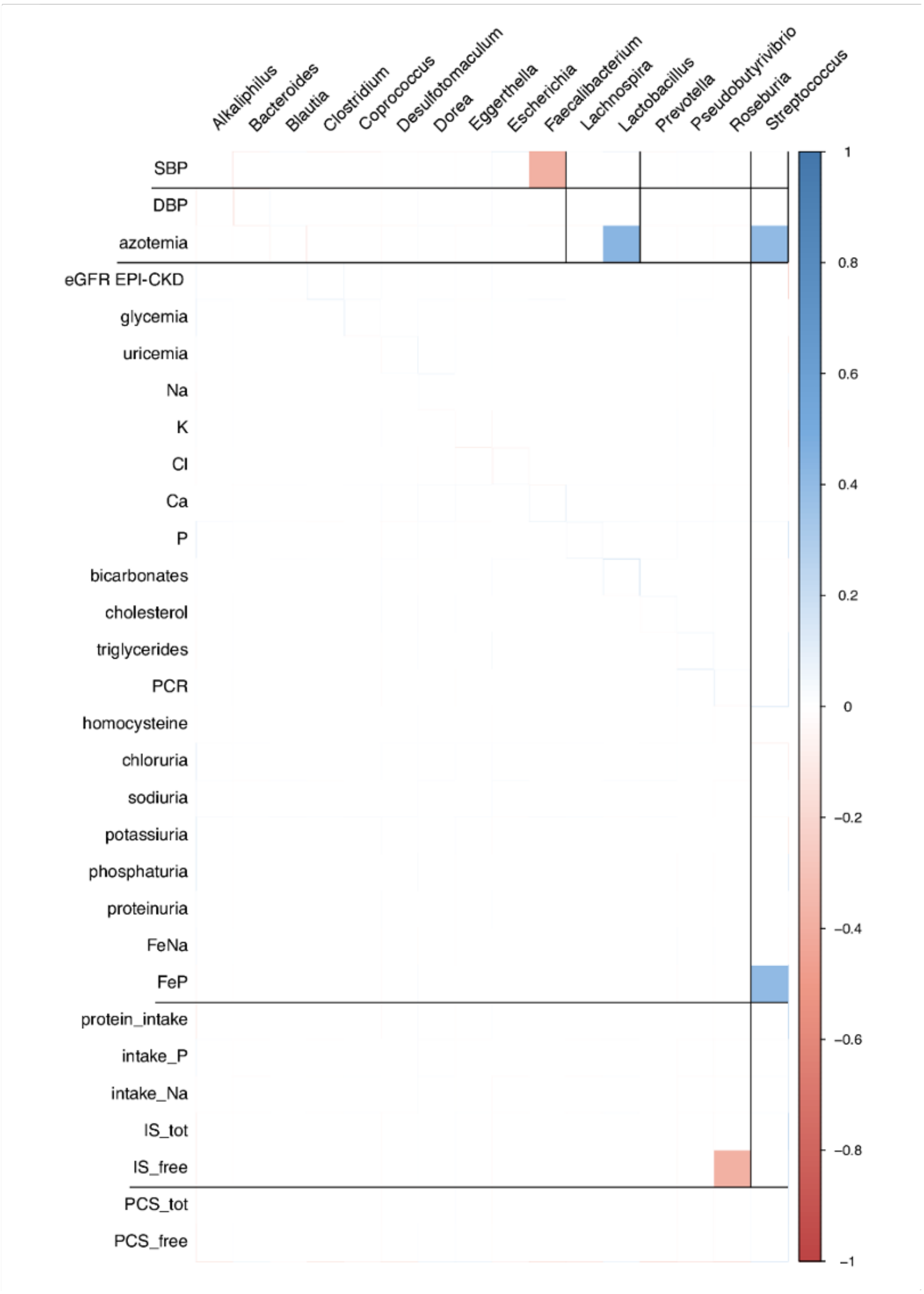

Supplement: Supplementary file 1 [file jcm-10-00840-s001.pdf]
